# A Case of Influenza B and *Mycoplasma pneumoniae* Coinfection in an Adult

**DOI:** 10.1155/2018/3529358

**Published:** 2018-11-25

**Authors:** Taro Shinozaki, Kotaro Sasahara, Eri Iwami, Aoi Kuroda, Tatsu Matsuzaki, Takahiro Nakajima, Takeshi Terashima

**Affiliations:** Department of Respiratory Medicine, Tokyo Dental College Ichikawa General Hospital, 5-11-13, Sugano, Ichikawa, Chiba 272-0824, Japan

## Abstract

A 19-year-old woman was referred to our hospital because of a persistent fever and cough that lasted for over a week. Influenza B virus infection was diagnosed using the rapid test kit. Initially, the patient was diagnosed with influenza B infection associated with lobar pneumonia and treated with an anti-influenza virus drug and sulbactam/ampicillin. The patient's fever persisted, and her respiratory condition worsened. On day 5, a computed tomography (CT) scan revealed an extension of the consolidation areas in the left lung and new opacities in the right lung. The antibiotic treatment was changed to meropenem and levofloxacin, and the patient's physical condition gradually improved. A sputum sample revealed the presence of *Mycoplasma pneumoniae*-specific DNA. Both influenza B virus and *M. pneumoniae* infections were confirmed serologically. This was a case of coinfection with influenza B virus and *M. pneumoniae* in a healthy young woman. The *M. pneumoniae* pneumonia diagnosis was delayed because the predominant feature observed in the CT scan was dense consolidation. *M. pneumoniae* should be considered as one of the causative pathogens in influenza coinfection cases with CT scan images presenting dense consolidation.

## 1. Introduction

Viral and bacterial coinfection is associated with increasing hospital admissions and more severe symptoms. The most common coinfecting species detected with influenza viruses are *Streptococcus pneumoniae*, *Staphylococcus aureus*, and *Haemophilus influenzae* [1–3]. In this report, we describe a case of influenza B virus and *Mycoplasma pneumoniae* coinfection in a healthy young woman. The predominant feature observed by computed tomography (CT) was dense consolidation, which is atypical for *M. pneumoniae* pneumonia.

## 2. Case Presentation

A 19-year-old woman was referred and admitted to our hospital because of a progressive fever and persistent cough. The patient was a university student who lived with her parents, brother, and grandmother. Her medical history was uninformative regarding risk factors; the patient had no smoking history. She had not received influenza vaccination during the season. Six days prior to admission, she experienced fever and visited a clinic. There had been an outbreak of influenza A and B virus infections in the area during that time. Aside from this, she had no episodes of exposure to pathogens causing acute fever. The nasopharyngeal swab sample was analyzed using a rapid test kit and did not indicate the presence of either type A or B influenza virus antigen. Repeated examination of a nasopharyngeal swab sample on the following day did not indicate any influenza virus antigen. Clarithromycin was administered based on a diagnosis of acute upper respiratory infection. As there was no clinical improvement in spite of five days of treatment, the patient was referred to our hospital.

At admission, a physical examination indicated that the patient was a well-developed and well-nourished woman. Her body temperature was 39.6°C, blood pressure was 108/65 mm Hg, pulse was 106 beats/min, respiratory rate was 24 breaths/min, and room air percutaneous oxygen saturation (SpO_2_) was 95%. The physical examination was unremarkable, and her respiratory sound was normal.

**Figure 1:**
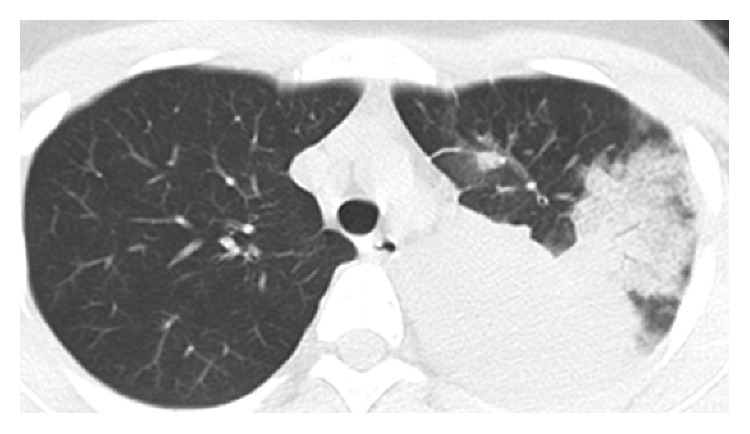
Chest computed tomography image on admission showing consolidation with an air bronchogram in the left upper lobe.

An initial laboratory examination showed a white blood cell count of 5,200/*µ*L (70% neutrophils), C-reactive protein level of 18.58 mg/dL, and procalcitonin level of 0.63 ng/mL (normal range <0.5 ng/mL). A nasopharyngeal swab sample analyzed using a rapid test kit (Quick Chaser Flu A, B; Mizuho Medy Co., Saga, Japan) indicated the presence of influenza B virus antigen. Stained sputum smears revealed Gram-positive cocci and Gram-negative rods. No pathogenic bacteria were cultured form repeated blood or sputum cultures. Chest radiography and CT scan (Figure 1) showed dense consolidation in the left upper lobe, indicating the presence of lobar pneumonia. The patient was diagnosed with influenza B virus infection accompanied by community-acquired pneumonia. The patient was treated with peramivir (600 mg/day) and sulbactam/ampicillin (SBT/ABPC; 12 g/day).

**Figure 2:**
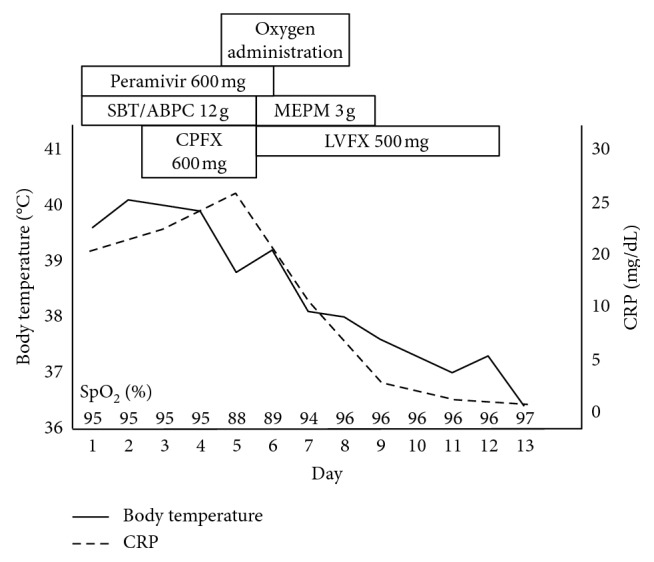
Clinical course of the patient. BT, body temperature; CPFX, ciprofloxacin; CRP, C-reactive protein; MEPM, meropenem; SBT/ABPC, sulbactam/ampicillin; SpO_2_, percutaneous oxygen saturation.

**Figure 3:**
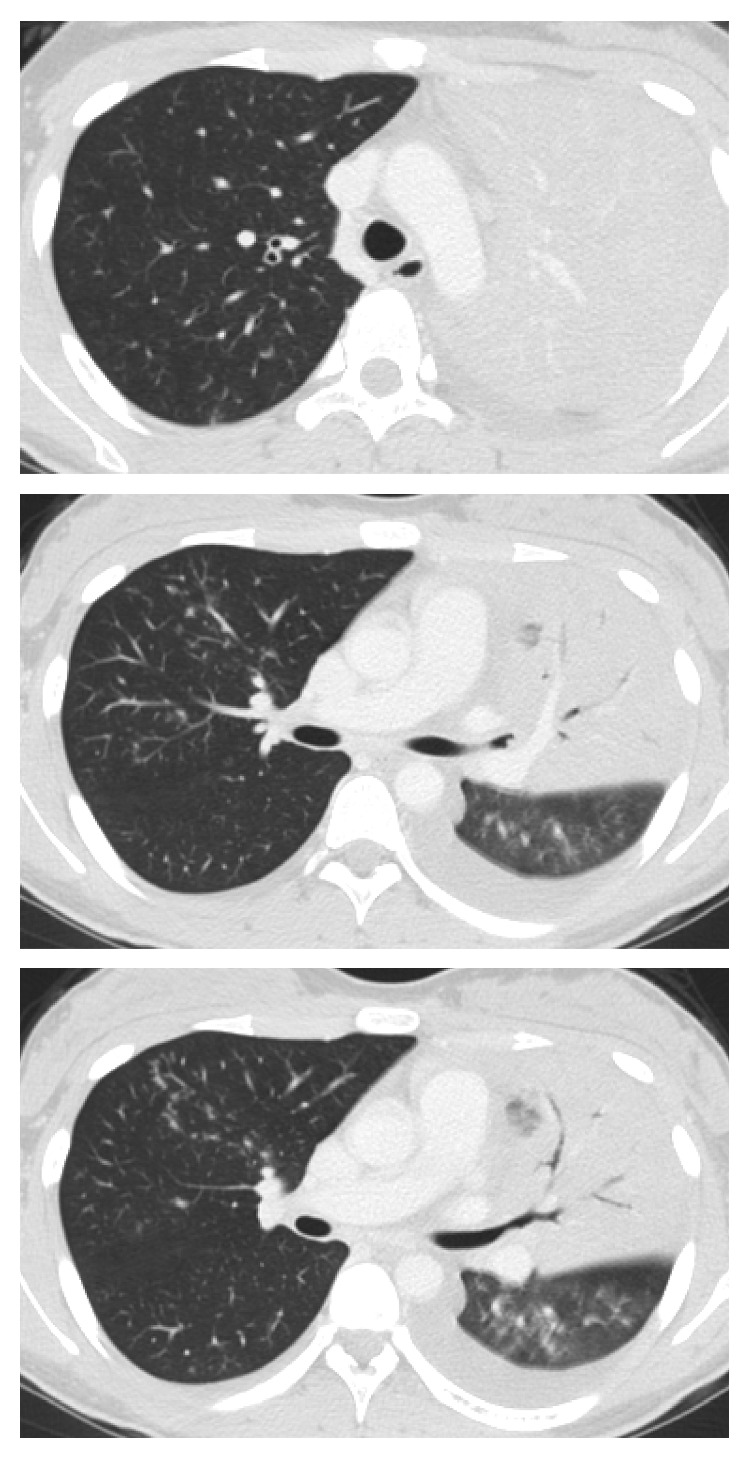
Chest computed tomography image on day 5 showing extension of the consolidation areas with air bronchogram in the left upper lobe and opacities in the right lung.

Following admission, the patient's fever persisted, and her respiratory condition worsened. The course of the patient's illness is shown in Figure 2. On day 3 after admission, her body temperature was 40.0°C, and a laboratory examination showed no improvement. Therefore, ciprofloxacin (CPFX; 600 mg/day) was added. On day 5, the high fever persisted, and the room air SpO_2_ was 88%. A chest CT scan revealed extension of the consolidation areas with air bronchogram in the left upper lobe and opacities in the right lung (Figure 3). On day 6, the SBT/ABPC and CPFX treatment was changed to meropenem (MEPM; 3 g/day) and levofloxacin (LVFX; 500 mg/day). On day 8, the patient's physical condition gradually improved, and the SpO_2_ recovered to >95% without oxygen administration. The sputum sample analyzed using a loop-mediated isothermal amplification assay (Mitsubishi Chemical Medience Co., Tokyo, Japan) revealed the presence of *M. pneumoniae*-specific DNA. There was no outbreak of *M. pneumoniae* infection in the area at the time. On day 13, the patient became afebrile and was discharged. The serum antibody titer against *M. pneumoniae* was 1 : 160 on admission and 1 : 10,240 on day 18 using the particle agglutination method and 1 : 32 on admission and 1 : 1024 on day 18 using the complement fixation test. In addition, the serum antibody titer against influenza B virus, measured using the complement fixation test, was <4 on admission and 1 : 128 on day 18. The serum antibody titer against influenza A virus did not increase throughout the clinical course.

## 3. Discussion

In this report, we describe a case of influenza B virus and *M. pneumoniae* coinfection in a previously healthy woman. Both influenza B virus and *M. pneumoniae* infections were confirmed serologically. To the best of our knowledge, there have been only a few reported cases of influenza B virus and *M. pneumoniae* coinfection [4]. The *M. pneumoniae* pneumonia diagnosis was delayed because the CT scan features were atypical and there was no outbreak of *M. pneumoniae* infection in the area.

According to a review by Brundage, *Staphylococcus aureus*, *Streptococcus pneumoniae*, and *H. influenzae* were the main pathogens associated with severe infection or death in pandemics that occurred in the twentieth century [3]. Several studies have investigated the pathogens associated with influenza coinfection. One study showed that the incidence of influenza B virus and *M. pneumoniae* coinfection was low [5]. Another study reported that of 11 patients with community-acquired pneumonia in whom influenza B virus was detected using PCR methods, four, four, and three patients were coinfected with *S. pneumoniae*, *Chlamydophila pneumoniae*, and *M. pneumoniae*, respectively [6]. However, these reports could be unique experiences and are not likely to be representative of all populations or locations. A meta-analysis conducted by Klein et al. indicated that the most common pathogens were *Streptococcus pneumoniae* and *Staphylococcus aureus*, which accounted for 35% and 28% of coinfections, respectively; a wide range of other infection-causing pathogens were identified including *Pseudomonas aeruginosa*, *Streptococcus pyogenes*, *H. influenzae*, *Klebsiella pneumoniae*, and *M. pneumoniae* [7]. Although the frequency of *M. pneumoniae* is lower than that of other bacterial pathogens, it should still be considered as one of the causative pathogens in influenza virus coinfections.

*M. pneumoniae* infection is acquired by inhalation of organisms, followed by an incubation period of 2–3 weeks. As the incubation period of the influenza virus is 1–3 days, we believe that the *M. pneumoniae* infection may have already existed in our patient's respiratory system at the time she was infected with influenza B virus. This hypothesis is supported by the fact that the serum antibody titer against *M. pneumoniae* was slightly elevated at the time of admission. Following adherence on the surface of bronchial cells, *M. pneumoniae* penetrates through the bronchial mucous membranes and releases nucleases and H_2_O_2_, which results in necrosis of bronchial epithelial cells and weakened cilia movement in the epithelium [8]. *M. pneumoniae* infection can induce immunosuppression in the body and cause dysfunction of cellular and humoral immunity [8]. It is possible that the patient's condition facilitated infection by influenza B virus because her systemic and local defense system had been impaired by *M. pneumoniae* pneumonia. Coinfection with influenza accounts for 9% of *M. pneumoniae* pneumonia cases that require hospitalization [9]. Alternatively, the influenza B virus infection was coincidental, as there was an outbreak of influenza A and B virus infections in the area during that time.

Additionally, it is possible that the dense consolidation and the opacities spread rapidly because the patient simultaneously suffered from influenza B infection. Viral damage to the epithelial lining of the respiratory tract is believed to facilitate the establishment and spread of other pathogens. Infection with influenza virus is thought to suppress the pulmonary epithelial immune system, which enables increased bacterial adherence and dissemination [10].

The commonly reported *M. pneumoniae* pneumonia CT findings are generalized bronchial wall thickening and peribronchial abnormal opacities, which are observed in 97% of cases [11]. In contrast, dense consolidation with air bronchogram is more frequent in community-acquired pneumonia due to pathogens other than *M. pneumoniae*. Macrolides are the first-line treatment for *M. pneumoniae* respiratory tract infections. However, macrolide resistance has been spreading, with prevalence ranging up to 50–90% in Japan [12]. Although an antibiotic sensitivity test was not performed, it is possible that the *M. pneumoniae* strain in our case was resistant to macrolides. Because the typical CT findings were absent in the initial CT image and no clinical improvement was achieved using clarithromycin, bacteria other than *M. pneumoniae* were suspected to be the causative pathogen, and the diagnosis was delayed. We believe that influenza B virus and *M. pneumoniae* coinfection cases occur more frequently than reported or generally appreciated. Although our case was one of a number of such instances, its clinical course and image features were unique and atypical. It should be noted that there have been other reports of cases with atypical clinical presentation similar to ours. In conclusion, *M. pneumoniae* should be considered as one of the causative pathogens in cases of influenza coinfection with CT scan images presenting dense consolidation.
